# Dexmedetomidine versus Remifentanil for Sedation during Awake Fiberoptic Intubation

**DOI:** 10.1155/2012/753107

**Published:** 2012-07-16

**Authors:** Davide Cattano, Nicholas C. Lam, Lara Ferrario, Carmen Seitan, Kash Vahdat, Darrell W. Wilcox, Carin A. Hagberg

**Affiliations:** ^1^Department of Anesthesiology, The University of Texas Medical School at Houston, Houston, TX 77030, USA; ^2^Department of Internal Medicine, Vanderbilt University, Nashville, TN 37240, USA; ^3^Department of Anesthesia, Duke University School of Medicine, Durham, NC 27710, USA

## Abstract

This study compared remifentanil and dexmedetomidine as awake fiberoptic intubation (AFOI) anesthetics. Thirty-four adult ASA I-III patients were enrolled in a double-blinded randomized pilot study to receive remifentanil (REM) or dexmedetomidine (DEX) for sedation during AFOI (nasal and oral). Thirty patients completed the study and received 2 mg midazolam IV and topical anesthesia. The REM group received a loading dose of 0.75 mcg/kg followed by an infusion of 0.075 mcg/kg/min. The DEX group received a loading dose of 0.4 mcg/kg followed by an infusion of 0.7 mcg/kg/hr. Time to sedation, number of intubation attempts, Ramsay sedation scale (RSS) score, bispectral index (BIS), and memory recall were recorded. All thirty patients were successfully intubated by AFOI (22 oral intubations/8 nasal). First attempt success rate with AFOI was higher in the REM group than the DEX group, 72% and 38% (*P* = 0.02), respectively. The DEX group took longer to attain RSS of ≥3 and to achieve BIS <80, as compared to the REM group. Postloading dose verbal recall was poorer in the DEX group. Dexmedetomidine seems a useful adjunct for patients undergoing AFOI but is dependent on dosage and time. Further studies in the use of dexmedetomidine for AFOI are warranted.

## 1. Introduction

Awake nasal or oral flexible fiberoptic intubation (AFOI) is usually the primary method for airway management in the *expected* difficult airway. Experience with AFOI is not easily acquired, and success of the procedure is also highly dependent on adequate preparation and sedation techniques, especially in training programs [1].

Optimal conditions for AFOI include that a patient be comfortable, cooperative, free of oropharyngeal blood and secretions, and able to maintain their airway with spontaneous ventilation. In order to achieve these conditions, the pharmacologic agent chosen for sedation should be short acting, easily titratable, provide the required amount of sedation and have little suppression of spontaneous ventilation. Controlled sedation and analgesia are paramount to AFOI, but deep sedation can result in loss of the airway with serious consequences. Techniques to improve success rate have included nasal over oral intubation (not always possible or not indicated in studies) and different protocols for sedation (sevoflurane, propofol and remifentanil with titrated or target controlled infusion) [1–9]. 

There have been numerous reports of remifentanil and propofol used either alone or in combination to achieve an adequate level of sedation for such procedures. The advantages of remifentanil for AFOI include the following: it is ultra-short acting with a constant half life, it has antitussive effects which help prevent coughing with tracheal manipulation, it is reversible with an antagonist, and finally, it attenuates cardiovascular responses to airway manipulation. The shortcomings of remifentanil include undesirable side effects, such as bradycardia and respiratory depression [1–9].

Dexmedetomidine is a centrally acting, selective alpha-2 agonist which has gained increasing popularity since 1999 as a drug for sedation in ICU settings [10, 11], for intraoperative sedation during surgery under regional anesthesia [12], for awake craniotomies [13], and for sedation of pediatric patients in different settings [14]. More recently, there have been several case reports of dexmedetomidine being used for AFOI [15–18]. 

Dexmedetomidine has been shown to have a rapid onset and equally rapid redistribution half life with quick recovery, it attenuates cardiovascular responses to laryngoscopy and intubation, thereby reducing the need for perioperative opioid and could have an amnestic effect [10–18].

Our study aim was to show at least equal efficacy in intubating conditions between Dexmedetomedine and remifentanil for both oral and nasal intubation, when the primary provider is a trainee anesthesiologist. Our primary outcome was to measure the time to sedation and the quality of intubating conditions. Our secondary outcome was to evaluate the number of attempts to secure the airway.

## 2. Material and Methods

During the years 2006 and 2007, after institutional review board approval from the University of Texas Medical School at Houston, written informed consent was obtained from 34 adult ASA I-III patients who required AFOI, as deemed necessary by the attending anesthesiologist. Due to case cancellations or delays, only 30 patients were included. These patients were randomized by the pharmacy into one of two groups: group REM (remifentanil) and group DEX (dexmedetomidine). Study drugs were prepared by the pharmacy in accordance to the patient's weight in kilograms and blinded to the anesthesia care team (faculty and resident) and the patient. All residents were CA-2 or CA-3 and had previously performed at least 5 oral and 5 nasal fiberoptic intubations. Their classification as “trainee” anesthesiologists provided the study with insight into how the “average” anesthesiologist (i.e., a mid-level provider not employed at a tertiary level hospital) would perform and which drug would be of more value to him. No stratification was decided between oral and nasal intubations.

The preparation of patients in each group was standardized as much as possible. After pretreatment with 0.2 mg IV glycopyrrolate and 2 mg IV midazolam, each patient was taken to the operating room where ECG, pulse-oximeter and a non-invasive blood pressure cuff were placed. A bispectral index (BIS) brain monitor (Aspect Medical, Norwood, MA, software revision 3.31) was also applied. Topical anesthesia and vasoconstrictor were applied depending on type of intubation. If oral intubation, the patient was placed in a semi-recumbent position and 2-3 mL lidocaine 4% was administered either via metered-atomization-device (MAD) catheter through the oral cavity and pharynx to reduce gag reflex or via oral cannula using Ovassapian fiberoptic inutubating airway. Following oral intubation, patients were placed in the supine position. If nasal intubation, the patient was placed in a supine condition (per institutional practice) and the vasoconstrictor oxymetazoline was sprayed in nose, followed by lidocaine 4% nebulized via MAD catheter. 

The preparation for and performance of awake intubation were standardized between the two groups. All patients received a loading dose at a rate of 0.1 mL/kg over 10 minutes and a continuous infusion at a rate of 0.1 mL/kg/hr of their respective drug via the Protégé 3010 Syringe Pump (Medex, Inc., Duluth, GA). Patients in group REM received a remifentanil loading dose of 0.75 mcg/kg (0.1 mL/kg at a concentration of 7.5 mcg/mL) and patients in group DEX received a dexmedetomidine loading dose of 0.4 mcg/kg (0.1 mL/kg at a concentration of 4 mcg/mL) over 10 minutes [3]. The continuous infusion was begun with patients in group REM receiving remifentanil at 0.075 mcg/kg/min (0.1 mL/kg/hr at a concentration of 45 mcg/mL) and those in group DEX receiving dexmedetomidine at 0.7 mcg/kg/hr (0.1 mL/kg/hr at a concentration of 7 mcg/mL) [3–19]. 

At that point, the anesthesiologist used the Ramsay sedation scale (RSS) to assess the level of sedation of the patient [20]. The RSS is a scale from 1–6 where 1 = agitated, 3 = responsive to commands only, and 6 = unresponsive. If the RSS was less than 3, up to 3 rescue doses at 1/4 the loading dose of the group's respective drug were administered. In both groups, the drug infusion was discontinued after successful intubation and induction of general anesthesia.

Baseline values were obtained using ECG, pulse oximeter (SpO_2_), systolic blood pressure (SBP), diastolic blood pressure (DBP), and bispectral monitor (BIS). Blood pressures were obtained every minute until intubation and then every 3 minutes thereafter. Level of sedation using the RSS was recorded every minute. Patient's blood pressure and heart rate (HR) were monitored and maintained during the procedure according to the following guidelines: SBP was maintained within 20% of the baseline value and HR was maintained within 20% of baseline. Incidents of hypertension, tachycardia, or bradycardia were recorded and treated accordingly. Crystalloid fluids (5–10 mL/kg) were administered during the loading phase of the drug. Episodes of apnea >60 seconds or a drop in O_2_ saturation <95% was treated by decreasing the infusion rate to 0.05 mL/kg/hr and bag mask ventilation with 100% oxygen, as necessary. For episodes of apnea longer than 2 minutes, infusion was discontinued and bag mask ventilation was commenced until the patient began to breathe spontaneously. After an additional 2 minutes, if the patient was in the REM group, naloxone was administered in 40 mcg doses IV every 1-2 minutes until spontaneous ventilation resumed. Once the patient started to breathe spontaneously, the infusion was restarted at 0.05 mL/kg/hr.

### 2.1. Memory Recall

For the purpose of recording the effect of the study drugs on memory recall, patients were shown pictures and spoken words for recall at various later times. Prior to the administration of any medication, a picture of a cat was shown and the word “apple” was spoken to all patients. After completion of the loading dose, a picture of scissors was shown and the word “tree” was spoken all patients. After completion of surgery and upon arrival to the PACU, a picture of a pen was shown and the word “boy” was spoken to all patients. At each instance, patients were asked to verbally confirm the picture and word and told that they would be asked to recall the items after surgery. Every 30 minutes until 180 minutes had transpired, each patient was asked to verbally recall the objects shown previously. If the patient did not recall the image immediately, the patient was shown a 4-item composite containing a picture of the image as one of the objects. If the patient still did not recollect the object after seeing the composite, the patient was considered to have no recall. In addition to recall of the visual items, recall of the spoken words was recorded. However, the patient was given no prompt if he or she did not recall the item immediately.

### 2.2. Statistical Analysis

Univariate and multivariate analyses were calculated with unpaired *t*-test, chi-square, Mann-Whitney, Cox regression, and Kaplan-Meyer analysis, as necessary. A *P* value <0.05 was considered significant. A power sample with an alpha of 0.05 and power of 0.8 established a sample size of 17 patients per group to find a difference of at least 30% reduction in number of events requiring dose reduction for oversedation and apnea with Dexmedetomedine. All calculations were performed with STATA (Stata Corp, v10, College Station, TX). 

Generalized linear mixed models (GLMMs) were calculated separately for the pre and postphysiological variables. Restricted maximum likelihood (REML) was used to estimate model parameters. The fixed or predictive component for all of the GLMMs included the drug group. The repeated outcome measurements were clustered within individual patients (a 2-level hierarchical model) to account for the correlations among measurements. Time of assessment was analyzed as a random variable in the calculated GLMMs. Four correlation structures accounting for the correlation among random effect parameters (the G matrix) were evaluated (identity, independent, exchangeable, and unstructured). The correlation structure for the error matrix (the R matrix) was the identity (diagonal) matrix. The identity correlation structure evaluated random intercept models; the other 3 structures were evaluated by random coefficient (random intercepts and slopes) models. Specifically, the individual patient differences in the physiological outcomes (random intercepts), as well as individual patient differences for changes in the physiological variables over time (random slopes) were evaluated by The Bayesian Information Criterion (BIC) to evaluate model fit. 

## 3. Results

Thirty randomized cases underwent awake fiberoptic intubation; 17 were in the REM group and 13 were in the DEX group. Unequal distribution resulted due to a prerandomized list that did not adjust for the four excluded DEX patients. AFOI was successful in all patients in both groups. Five patients in the REM group and 3 patients in the DEX group received nasal endotracheal intubation.

Table 1 demonstrates the advantages and disadvantages between remifentanil and Dexomedetomidine. There were no significant differences between the two groups with respect to age, Mallampati classification, ASA, BMI, and thyromental distance (Table 2).

In order to interpret the successful number of intubation attempts, ordered regression analysis was performed to adjust for difficult intubation (DI) using the variables and cut-off values that predict difficult intubation cases such as sternomental distance (<12.5 cm), thyromental distance (<6.5 cm), age (>55 yrs), Mallampati classification (>2), history of difficult intubation, BMI (>35 kg/m^2^), and inadequate neck mobility. Seventy-six percent of the REM group were intubated on the first attempt, as compared to 38% of the DEX group (*P* = 0.02). In both adjusted and unadjusted analysis, intubation attempts were greater for the DEX group (OR unadjusted = 5.26, 95% C.I. = 1.19–25.72; OR adjusted = 4.84, 3.43–6.82). DEX group had 3 nasal intubations and REM group had 5 nasal intubations which had no significant impact on the number of intubation attempts for either drug at an adjusted odds ratio of 5.51 (95% C.I. = 1.16–26.08) for type of intubation.

**Figure 1:**
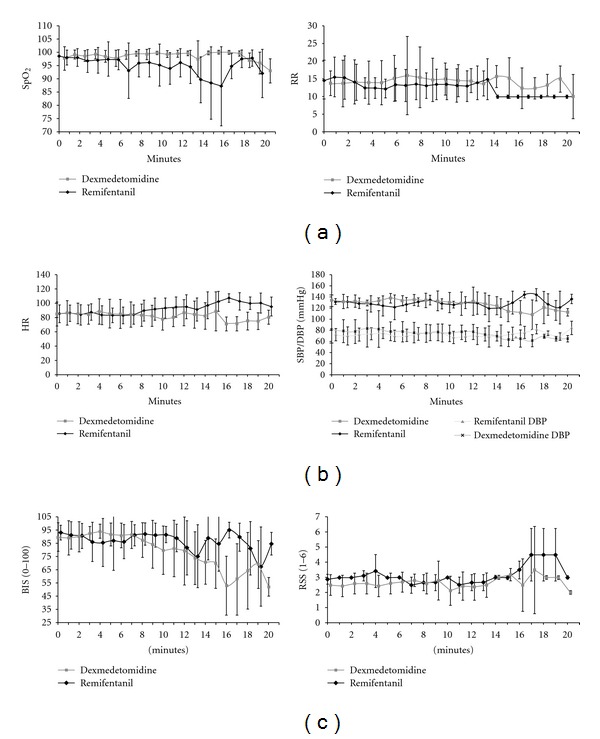
Mean physiologic variables from beginning to end of intubation. (a) includes oxygen saturation (SpO_2_) and respiratory rate (RR). (b) includes heart rate (HR) and systolic (SBP) and diastolic blood pressure (DBP). (c) includes sedation variables bispectral index (BIS) and Ramsay Sedation Scale (RSS).

Since the intubation period varied for each patient from 1–20 minutes in both groups, mean HR, SBP, DBP, SpO_2_, RR, BIS, and RSS were analyzed (Figure 1) in addition to GLMMs to account for the variations in the physiological measurements recorded (Table 3). Predicted means for REM and DEX effects were for a “typical” patient: a male with an ASA of II at time 0 with an adjusted difficulty intubation score of 0.46.

There were no statistically significant differences between the mean oxygen saturations and respiration rates when comparing the REM group and the DEX group. There was no statistically significant difference in the incidence of O_2_ saturation <90% between the two groups. In addition, no apneic episodes occurred and no rescue maneuvers were required in either group, such as administration of reversal drugs or positive pressure ventilation.

There was no appreciable significance between predicted means for REM and DEX groups with respect to HR (79.60 and 78.44), SBP (122.74 and 129.84), and DBP (75.33 and 76.41). Ten minute standard mean calculation of HR between the 2 groups showed no change. 

The DEX group had a lower predicted RSS mean score of 2.41 (2.10–2.71) compared to the REM group predicted RSS mean score of 2.88 (2.52–3.24). Thus, the DEX group predicted RSS mean score was significantly lower by 0.47 (95% C.I. = 0.17–0.78; *P* = 0.002). The main time effect for the RSS score was significant, but the drug to time interaction showed no significance. A Kaplan Meier survival analysis was also calculated to contrast the time to a RSS score of ≥3. Drug differences were significant (Logrank test = 4.00 with 1 degree of freedom, *P* = 0.0455). The DEX patients took longer to attain an RSS score of ≥3 than the REM patients. After 1 minute, almost all the REM patients had an RSS score of ≥3, while only half the DEX patients had an RSS of ≥3 at 5 minutes. 

BIS predicted means for a “typical” REM patient = 87.56 (81.62–93.49) and a “typical” DEX patient = 88.19 (82.99–93.40). A Kaplan Meier survival analysis was also calculated to contrast the time to a BIS <80. Drug differences approached significance (Logrank test = 3.25 with one degree of freedom, *P* = 0.0715). REM patients attained a BIS <80 sooner than the DEX patients. At 10 minutes, 68% (11) of the REM versus 39% (6) of the DEX patients had a BIS <80. Half of the REM patients had a BIS <80 after 5 minutes, while it was not until 13 minutes that half the DEX patients had a BIS <80. A Cox regression was calculated to adjust drug differences by gender, ASA, and DI differences. After adjusting for those covariates, the drug differences were significant (chi-square = 4.987 with 1 d.f., *P* = 0.026; hazard ratio = 3.518 (1.094–11.316)). 

Recall scores used generalized estimating equations to evaluate the significance of the recall results and significance was only observed in postloading dose verbal recall of scissors. Postloading dose verbal recall was poorer for DEX group after adjusting for ASA, difficult intubation, and gender (OR = 0.25, 95% C.I. = 0.06–1.00; *P* = 0.05).

No serious complications occurred in either group throughout the awake intubation procedures. Three REM cases required intervention for tachycardia and hypertension and one for inadequate sedation, while 4 DEX cases required intervention for tachycardia, inadequate sedation, hypertension, and hypotension (Table 4). Upon postoperative assessment, 3 patients in the REM group remembered that the fiberscope was in their mouth, and one patient claimed to have experienced pruritus of their nose and eyes during the intubation procedure, whereas 2 patients in the DEX group remembered that the fiberscope was in their mouth.

## 4. Discussion 

The current study showed relatively similar efficacy of Dexemedetomidine and remifentanil as adjuvant to endotracheal oral and nasal intubation. The study ended being underpowered by 4 patients in the DEX group.

In this study, patients who received dexmedetomidine and remifentanil were sufficiently sedated with similar hemodynamic profiles for successful AFOI. Nonetheless, we found that the patients in the DEX group had an increased number of intubation attempts and delayed intubation start time, possibly due to the following: lower dexmedetomidine loading dose, different mechanism of sedation between the two agents, greater analgesic inhibition of airway reflexes by remifentanil, and time differential of sedation assessment between the two groups. Compared to other studies, the sedation score chosen was averagely 1-2 points lower on the RSS. The dosages used of remifentanil and dexmedetomidine were also different [1–8, 14–18].

Creating an appropriate sedation state for a patient for any given situation is not an exact science. Additionally, the optimum sedation dose for dexmedetomidine for AFOI has not been established, although a loading dose of 0.4 mcg/kg to 1 mcg/kg over 10 minutes and beyond has been used to attain sedation. In the present study, a relatively low loading dose of 0.4 mcg/kg over 10 minutes was used, followed by a higher infusion rate of 0.7 mcg/kg/hr. Additionally, 2 mg IV midazolam was administered to patients in both groups to provide amnesia. As a result of the lower range of loading dose used, no appreciable changes in hemodynamics occurred in the DEX group. However, this loading dose might have resulted in insufficient sedation and analgesia for a successful first attempt at awake fiberoptic intubation. It is difficult to compare our study protocol (considering the challenges of the blind randomization), where we utilized multilevel sedation/anesthetic with other published works.

Jaakola et al. [19] evaluated dexmedetomidine and fentanyl at various doses in healthy volunteers and concluded that moderate analgesic properties are reached at approximately 0.5 mcg/kg, slightly greater than the loading dose in the current study. The optimum drug dose for a sedative to achieve a careful balance of airway relaxation versus collapse is difficult to ascertain. A study performed by Hall et al. [21] assessed the patient's alertness every 10 minutes, as opposed to 1-minute intervals. Remifentanil was scrutinized the same way and it achieved an RSS score of 3 almost immediately after the loading dose, therefore, attempts at AFOI were begun earlier than when dexmedetomidine was used. Dexmedetomidine achieved an RSS score of 3 at a slower rate than remifentanil, but it always achieved enough sedation to begin AFOI. Half the REM patients had a BIS <80 after 5 minutes, while it was not until 13 minutes that half the DEX patients had a BIS <80. This does not necessarily explain why once an RSS score of 3 was achieved, there were still more attempts needed to successfully intubate a patient in the DEX group. However, this delay in sedation allowed the DEX patients to remain at awake levels longer, leading to more stimulation from the ongoing RSS assessments every minute, while the REM group did not continuously undergo RSS assessments because almost all REM patients achieved RSS scores of 3 or greater immediately postloading dose.

Although a low loading dose of dexmedetomidine was well tolerated in this study, additional reports have recently described success with a higher loading dose, either alone or in combination with other agents including midazolam and ketamine. In fact, Scher and Gitlin [15] not only used 1 mcg/kg dexmedetomidine, but also added 15 mg ketamine bolus followed by an infusion of 20 mg/hr to achieve excellent intubating conditions for AFOI, including satisfactory sedation, patient cooperation, and a dry airway. While benzodiazepines have been shown to have a synergistic effect with dexmedetomidine [22, 23], to our knowledge, all studies involve animals, and clinical trials have yet to verify this synergism and the dosage required to achieve it. 

As low as the loading does of dexmedetomidine was, the memory recall results indicate that dexmedetomidine has a significantly stronger amnestic effect than remifentanil. As our study acquainted patients with words and pictures and asked for their confirmation preoperatively, our findings support the theory that sedation-independent memory impairment interferes with the retention of successfully acquired information, as opposed to interfering with the acquisition of new material as is the effect of benzodiazepines [24].

There were several limitations in this study. First and foremost, the pilot nature of the study requires that its results be viewed with caution and studies using larger groups are required to confirm the findings. The inclusion of two different routes for accessing the airway (i.e., nasal and oral intubation) also creates issues as not all patients received the same type of intubation. Lastly, as mentioned before, this study used different dosages of dexmedetomidine and remifentanil than was used in other studies so a direct comparison is not feasible. This study was conducted at a time when not much was known about the clinical efficacy or the pharmacokinetics of dexmedetomidine. Likewise, the dosages used were appropriate for the known effects of dexmedetomidine and standards per manufacturer recommendations. Any adjustment in the dosage given of dexmedetomidine will need to keep in mind possible side effects. While the seniority of the study is a limitation, the study still provides clinically sound information that is of use to anesthesia providers.

As our study is aimed at evaluating the value of dexmedetomidine for AFOI for the mid-level provider, further controlled clinical trials are warranted to investigate the use of a higher loading dose of dexmedetomidine for AFOI, specifically the dose which will predict a 90% success rate for AFOI on the first attempt.

## 5. Conclusion

Mid-level providers of anesthesia will find it of more benefit to use remifentanil for awake fiberoptic intubation.

## Figures and Tables

**Table 1:** Advantages and disadvantages of remifentanil versus dexmedetomidine.

Remifentanil	*Advantages*	*Disadvantages*
	Sedative	Hemodynamic instability
	Analgesic	Respiratory depression
	Ultra-short acting	
	Anti-tussive	
	Reversed by naloxone	

Dexmedetomidine	*Advantages*	*Disadvantages*
	Sedative	High cost ($80 for 200 mcg vial)^∗^
	Analgesic	Need for slow controlled bolus
	Xerostomia	followed by titrated infusion
	Minimal respiratory depression	Limited availability in European countries

^∗^Cost at Memorial Hermann Hospital, Houston, TX.

**Table 2:** Patient demographics between the two groups.

	Remifentanil (REM)	Dexmedetomidine (DEX)	*P* value
Number of patients	17	13	
Age (yrs)	50.3 ± 15.9	49.5 ± 14.9	0.89
Mallampati (I, II, III, IV) *n *(%)	1/5/9/2 (8%, 31%, 54%, 15%)	4/5/1/3 (30%, 40%, 10%, 20%)	0.15
ASA (1, 2, 3) *n *(%)	1/14/2 (5%, 83%,12%)	0/4/9 (0%, 31%, 69%)	0.96
Height (cm)	174 ± 10	171 ± 8	0.43
Weight (kg)	92 ± 19	76 ± 29	0.04
BMI (kg/m^2^)	30 ± 6	26 ± 8	0.13
Thyromental (cm)	7.2	6.1	0.08

**Table 3:** Physiologic data of patients in the two groups. Data includes heart rate (bpm), respiratory rate (bpm), oxygen saturation (%), systolic blood pressure (mmHg), bispectral index level, and Ramsay sedation scale score.

	Remifentanil (REM)	Dexmedetomidine (DEX)
Heart rate (bpm)	89.15 ± 14.38	84.71 ± 16.86
Respiratory rate (bpm)	13.55 ± 4.66	14.56 ± 6.04
Oxygen saturation (%)	95.95 ± 6.63	98.82 ± 6.63
Systolic blood pressure (mmHg)	124.4 ± 20.89	130.1 ± 25.89
Bispectral index level	87.38 ± 15.43	83.05 ± 18.01
Ramsay sedation scale score	3.06 ± 0.73	2.60 ± 0.84

There were no statistically significant differences identified.

**Table 4:** Adverse effects observed in patients between the two groups. Data includes intubation attempts, desaturation (SpO_2_ < 90%), hypotension, hypertension, bradycardia, and tachycardia.

	Remifentanil (REM)	Dexmedetomidine (DEX)
Intubation Attempts (1 : 2 : 3); *n* (%)	13 (76%), 3 (18%), 1 (6%)	5 (38%), 4 (31%), 4 (31%)
Desaturation ( SpO_2_ < 90%)	6 (35%)	2 (15%)
Hypotension (SBP < 90 mmHg)	2 (12%)	2 (15%)
Hypertension (SBP < 180 mmHg)	1 (6%)	2 (15%)
Bradycardia (HR < 40 bpm)	2 (12%)	1 (8%)
Tachycardia (HR > 100 bpm)	6 (35%)	7 (53%)

There are no statistical significant differences between the 2 groups.
